# Supplementary material for: Progress towards controlling the HIV epidemic in urban Ethiopia: Findings from the 2017–2018 Ethiopia population-based HIV impact assessment survey
Source: PLoS One. 2022 Feb 25;17(2):e0264441. doi: 10.1371/journal.pone.0264441 (PMC8880883; doi:10.1371/journal.pone.0264441)
Supplement: Attachment — Submitted filename: Comments_ICAP Ethopia.pdf [file pone.0264441.s002.pdf]

The authors have done a commendable Job and addressed all my comments satisfactorily. I congragulate them upon this. I have a few minor comments here below.

Line 42 to line 45: “Awareness Females ….” Statement not clear, review and correct

Line 77:”… prevalence of HIV infection in all aged,” replace aged with “age groups”

Line 85: “… health survey (EDHS) was supplemented by ….” Replace with “… health survey (EDHS), supplemented by ….”

Line 321: “…the implication high risk …” replace with “….the implication of high risk…”

Line 305; “… 79% knew that they were HIV positive status” drop the word status

Line 345- 350: the conclusion on the paragraph does not reflect the result in the paragraph, please review. i.e if age and gender were not statistically significant, what is the basis of targeting young people and men as you have stated in the paragraph
