# Supplementary material for: Progress towards controlling the HIV epidemic in urban Ethiopia: Findings from the 2017–2018 Ethiopia population-based HIV impact assessment survey
Source: PLoS One. 2022 Feb 25;17(2):e0264441. doi: 10.1371/journal.pone.0264441 (PMC8880883; doi:10.1371/journal.pone.0264441)
Supplement: Attachment — Submitted filename: Response to Reviewers.docx [file pone.0264441.s001.docx]

PLOS ONE Decision: Revision required [PONE-D-21-26375] - [EMID:0acdc77b9e65a587]

PONE-D-21-26375

Progress towards controlling the HIV epidemic in urban Ethiopia: Findings from the 2017-2018 Ethiopia population-based HIV impact assessment survey

PLOS ONE

Dear Dr. Lulseged,

Thank you for submitting your manuscript to PLOS ONE. After careful consideration, we feel that it has merit but does not fully meet PLOS ONE’s publication criteria as it currently stands. Therefore, we invite you to submit a revised version of the manuscript that addresses the points raised during the review process.

Both reviewers agree on the significant contribution this work will make on HIV policy.  However, they have also both identified significant issues that must be addressed in order for this work to advance at PLOS ONE.  Importantly, I note that Reviewer #1 answered "No" to "Has the statistical analysis been performed appropriately and rigorously?" and Reviewer #2 answered "Partly" and "No" to the questions "Is the manuscript technically sound, and do the data support the conclusions?" and "Is the manuscript presented in an intelligible fashion and written in standard English?", respectively. At PLOS ONE, a "Yes" to each of these items is required.

While the authors must address all reviewer comments, I would like to draw special attention to the following issues.  Both reviewers found significant issues with grammar, sentence structure and typos and feel the manuscript requires a thorough copy-edit to improve clarity.  Reviewer #1 raises important concerns about the need for statistical analyses that include the simultaneous effects of multiple variables. The reviewers have both commented on the fact that the surveys focused on urban areas. While I agree that this may not be a major limitation (as Reviewer #1 feels), I do agree with Reviewer #2 that this fact should be introduced clearly and earlier in the manuscript so the reader can make their own assessment of this aspect.

Please submit your revised manuscript by Dec 18 2021 11:59PM. If you will need more time than this to complete your revisions, please reply to this message or contact the journal office at [plosone@plos.org](mailto:plosone@plos.org). When you're ready to submit your revision, log on to <https://www.editorialmanager.com/pone/> and select the 'Submissions Needing Revision' folder to locate your manuscript file.

Please include the following items when submitting your revised manuscript:

- A rebuttal letter that responds to each point raised by the academic editor and reviewer(s). You should upload this letter as a separate file labeled 'Response to Reviewers'.
- A marked-up copy of your manuscript that highlights changes made to the original version. You should upload this as a separate file labeled 'Revised Manuscript with Track Changes'.
- An unmarked version of your revised paper without tracked changes. You should upload this as a separate file labeled 'Manuscript'.

If you would like to make changes to your financial disclosure, please include your updated statement in your cover letter. Guidelines for resubmitting your figure files are available below the reviewer comments at the end of this letter.

If applicable, we recommend that you deposit your laboratory protocols in [protocols.io](http://protocols.io/) to enhance the reproducibility of your results. Protocols.io assigns your protocol its own identifier (DOI) so that it can be cited independently in the future. For instructions see: <https://journals.plos.org/plosone/s/submission-guidelines#loc-laboratory-protocols>. Additionally, PLOS ONE offers an option for publishing peer-reviewed Lab Protocol articles, which describe protocols hosted on [protocols.io](http://protocols.io/). Read more information on sharing protocols at <https://plos.org/protocols?utm_medium=editorial-email&utm_source=authorletters&utm_campaign=protocols>.

We look forward to receiving your revised manuscript.

Kind regards,

David Gerberry, Ph.D.
Academic Editor
PLOS ONE

Journal Requirements:

When submitting your revision, we need you to address these additional requirements.

1. Please ensure that your manuscript meets PLOS ONE's style requirements, including those for file naming. The PLOS ONE style templates can be found at 
<https://journals.plos.org/plosone/s/file?id=wjVg/PLOSOne_formatting_sample_main_body.pdf> and 
<https://journals.plos.org/plosone/s/file?id=ba62/PLOSOne_formatting_sample_title_authors_affiliations.pdf>

**Authors’ Response**: Double-checked to ensure that the manuscript meets PLOS ONE's style requirements, including the file naming. As suggested, a thorough copy-edit of the manuscript has been made to improve clarity, statistical analyses showing simultaneous effects of multiple variables included and description added in all relevant sections of the manuscript, and the urban focus of the survey is now introduced clearly and earlier in the manuscript. More details are given below under specific comments by the reviewers.

2. In the Methods section of your manuscript, please provide additional information regarding the criteria used to determine emancipation of minors. Furthermore, please could you clarify for minors ages between 15-18, who were not considered emancipated, whether you obtained consent from parents or guardians of the minors included in the study or whether the research ethics committee or IRB specifically waived the need for their consent

**Authors’ Response**: The definition of ‘emancipated minor’ is now clearly provided, and a reference included. Parental/Guardian permission and child’s assent are included for non-emancipated children 15-17 years of age**.**

3. Please include additional information regarding the survey or questionnaire used in the study and ensure that you have provided sufficient details that others could replicate the analyses. For instance, if you developed a questionnaire as part of this study and it is not under a copyright more restrictive than CC-BY, please include a copy, in both the original language and English, as Supporting Information

**Authors’ Response**: More information on the survey questionnaire is provided in the manuscript. Reference (website) is also added for those interested to access the full complement of the original version (which is in English) of the survey questionnaire.

4. Please update your submission to use the PLOS LaTeX template. The template and more information on our requirements for LaTeX submissions can be found at <http://journals.plos.org/plosone/s/latex>

**Authors’ Response**: The submission is updated for use of the PLOS LaTeX template

5. We note that Figure(s) Appendix 1, 2 and 3 in your submission contain [map/satellite] images which may be copyrighted. All PLOS content is published under the Creative Commons Attribution License (CC BY 4.0), which means that the manuscript, images, and Supporting Information files will be freely available online, and any third party is permitted to access, download, copy, distribute, and use these materials in any way, even commercially, with proper attribution. For these reasons, we cannot publish previously copyrighted maps or satellite images created using proprietary data, such as Google software (Google Maps, Street View, and Earth). For more information, see our copyright guidelines: <http://journals.plos.org/plosone/s/licenses-and-copyright>.
 
We require you to either (1) present written permission from the copyright holder to publish these figures specifically under the CC BY 4.0 license, or (2) remove the figures from your submission:
 
1. You may seek permission from the original copyright holder of Figure Appendix 1, 2 and 3 to publish the content specifically under the CC BY 4.0 license.  
 
We recommend that you contact the original copyright holder with the Content Permission Form (<http://journals.plos.org/plosone/s/file?id=7c09/content-permission-form.pdf>) and the following text:
“I request permission for the open-access journal PLOS ONE to publish XXX under the Creative Commons Attribution License (CCAL) CC BY 4.0 (<http://creativecommons.org/licenses/by/4.0/>). Please be aware that this license allows unrestricted use and distribution, even commercially, by third parties. Please reply and provide explicit written permission to publish XXX under a CC BY license and complete the attached form.”
 
Please upload the completed Content Permission Form or other proof of granted permissions as an "Other" file with your submission.
 
In the figure caption of the copyrighted figure, please include the following text: “Reprinted from [ref] under a CC BY license, with permission from [name of publisher], original copyright [original copyright year].”
 
2. If you are unable to obtain permission from the original copyright holder to publish these figures under the CC BY 4.0 license or if the copyright holder’s requirements are incompatible with the CC BY 4.0 license, please either i) remove the figure or ii) supply a replacement figure that complies with the CC BY 4.0 license. Please check copyright information on all replacement figures and update the figure caption with source information. If applicable, please specify in the figure caption text when a figure is similar but not identical to the original image and is therefore for illustrative purposes only.
The following resources for replacing copyrighted map figures may be helpful:
 
USGS National Map Viewer (public domain): <http://viewer.nationalmap.gov/viewer/>
The Gateway to Astronaut Photography of Earth (public domain): <http://eol.jsc.nasa.gov/sseop/clickmap/>
Maps at the CIA (public domain): <https://www.cia.gov/library/publications/the-world-factbook/index.html> and <https://www.cia.gov/library/publications/cia-maps-publications/index.html>
NASA Earth Observatory (public domain): <http://earthobservatory.nasa.gov/>
Landsat: <http://landsat.visibleearth.nasa.gov/>
USGS EROS (Earth Resources Observatory and Science (EROS) Center) (public domain): [http://eros.usgs.gov/#](http://eros.usgs.gov/)
Natural Earth (public domain): <http://www.naturalearthdata.com/>

**Authors’ Response**: These figures do not add much. We have opted to removing Figures 5,6 and 7 (Appendices 1, 2 and 3) from the submission. Sections referring to the appendices are revised..

Please review your reference list to ensure that it is complete and correct. If you have cited papers that have been retracted, please include the rationale for doing so in the manuscript text, or remove these references and replace them with relevant current references. Any changes to the reference list should be mentioned in the rebuttal letter that accompanies your revised manuscript. If you need to cite a retracted article, indicate the article’s retracted status in the References list and also include a citation and full reference for the retraction notice.

**Authors’ Response**: All checked carefully for completeness and accuracy. No retracted reference is used.

Given the comments/suggestions given by the reviewers, which quite rightly and usefully required a major revision to the manuscript, we have made major edits and re-written some sections. We have expanded some sections by adding more information. We have also added a regression analysis model along with the description required in the different sections of the manuscript. We have reorganized the discussion section taking into consideration the results of the multivariate analysis focusing on discussing the results vis a vis their implications to the program, as rightly advised by the reviewers.

Removed ref#8 on Tuberculosis Burden in Ethiopia; cited by error and Ref#10 moved here instead.

Order of references reorganized with the revision made in the text.

References added with revision in the text

- #9 on Barriers and facilitators of adherence
- #17 on jackknife estimate of variance
- #19 on Ethiopian Research Ethics Guideline
- #25 on knowledge and condom use
- #27 on risk Behaviour of HIV Positive Individuals
- #28 on barriers to target achievement
- #29 on HIV status awareness and linkage to care
- #30 on higher risk sexual behaviour
- #34 on Barriers to HIV testing
- #39 on assessing progress towards the 2020 target
- #40 on quantifying the contribution of different aged men
- #42 on Sociodemographic factors affecting viral load suppression
- #43 on factors associated with unsuppressed viral load
- #44 on status of HIV/AIDS in the ART era
  .

[Note: HTML markup is below. Please do not edit.]

Reviewers' comments:

Reviewer's Responses to Questions

**Comments to the Author**

1. Is the manuscript technically sound, and do the data support the conclusions?

The manuscript must describe a technically sound piece of scientific research with data that supports the conclusions. Experiments must have been conducted rigorously, with appropriate controls, replication, and sample sizes. The conclusions must be drawn appropriately based on the data presented.

Reviewer #1: Yes

Reviewer #2: Partly

2. Has the statistical analysis been performed appropriately and rigorously?

Reviewer #1: No

Reviewer #2: Yes

3. Have the authors made all data underlying the findings in their manuscript fully available?

The [PLOS Data policy](http://www.plosone.org/static/policies.action#sharing) requires authors to make all data underlying the findings described in their manuscript fully available without restriction, with rare exception (please refer to the Data Availability Statement in the manuscript PDF file). The data should be provided as part of the manuscript or its supporting information or deposited to a public repository. For example, in addition to summary statistics, the data points behind means, medians and variance measures should be available. If there are restrictions on publicly sharing data—e.g. participant privacy or use of data from a third party—those must be specified.

Reviewer #1: Yes

Reviewer #2: Yes

4. Is the manuscript presented in an intelligible fashion and written in standard English?

PLOS ONE does not copyedit accepted manuscripts, so the language in submitted articles must be clear, correct, and unambiguous. Any typographical or grammatical errors should be corrected at revision, so please note any specific errors here.

Reviewer #1: Yes

Reviewer #2: No

5. Review Comments to the Author

Please use the space provided to explain your answers to the questions above. You may also include additional comments for the author, including concerns about dual publication, research ethics, or publication ethics. (Please upload your review as an attachment if it exceeds 20,000 characters)

Reviewer #1: The authors analyzed data from the Ethiopian Population HIV Impact Assessment conducted in 2017/18 to assess the progress made towards attaining the UNAIDS- 90-90-90 targets and the associated socio-demographic and behavioural factors in Urban Ethiopia. They found that 79.0% of the respondents infected by HIV were aware of their HIV status based on self-report or detection of ARV metabolites; 97.1% of those aware, were on antiretroviral therapy; and of those on treatment, 87.6% had achieved viral suppression. Some disparities by population subgroups were found in achieving the first 90, while no disparities were found for the second and third 90 targets.
The authors’ findings are informative and will guide program implementation efforts in urban areas in Ethiopia and in other countries in Sub Saharan Africa.
I have the following general comments:

1. There are some typos in the abstract and in the main document, the authors may consider reading through carefully and correct them including sentence structure. I have highlighted some examples here below.

**Authors’ Response**: Typos corrected, and sentence structure revised where required.

2. Analysis for the associated socio-demographic and behavioural factors have been limited to bivariate level, hence no consideration of simultaneous effect of multiple variables – which is a more realistic representation of our environments, the conclusions drawn here may not therefore be realistic to the rea real-life situation. Additionally, the authors have previously published a paper “Factors associated with unawareness of HIV positive status in urban Ethiopia ….” in which the correct analysis approach was followed.

**Authors’ Response**: A multiple regression analysis is done, and the results summarized in a Table. Text related to this is added, as appropriate, in the different sections of the manuscript.

3. More detailed explanation of the survey design; field processes such as consenting of survey participants; and ethical considerations are already published in the survey report. The authors could focus more on analysis methods for the first and second objectives and in discussing the implication of the analysis results to program implementation.

**Authors’ Response**: Cross-referencing is made to the published survey report. Only highlights of the survey design included. More details are added in the data analysis section, including the statistics used added in the methods (both in the abstract and the in body of the manuscript).

4. In the appendices, figures/maps are presented but there is no description of analysis methods used and linkage to the objectives of the study.

**Authors’ Response**: as explained above, Figures 5,6 and 7 (Appendices 1, 2 and 3) are deleted from the manuscript. Sections referring to the appendices are revised.

5. Limitations, please add more. Focusing the survey in urban areas, I think may not qualify as a major limitation. The authors have rightly noted in the conclusions, these are the most affected sub areas which require more intensified efforts in the absence of adequate resources.

**Authors’ Response**: Focus on urban areas is emphasized upfront in the methods section (under *study setting and population*). This is indicated in the limitation section with a tempered language.

6. Methods of analysis is not explicitly stated in the methods section of the abstract. This will be helpful to link the results to the methods and the conclusions drawn.

**Authors’ Response**: The methods of analysis is now added in the methods section of the Abstract, including the regression models add in this revised version.

Additional specific comments
- Line 63: “… however, with the end of 2020, ….” Review and revise the statement

**Authors’ Response**: Revised the statement (line 71).

- Review and correctly restate the statement of targets. It does not read correct --Line 67: should read “…. 95% of PLHIV who know their status are on treatment”

**Authors’ Response**: Restated (lines 68-69).

- Line 68: “…..have virally suppressed” replace with “…. have suppressed viral loads”

**Authors’ Response**: Replaced (line 69).

- Line 181-182 “…62% did not use a condom the last sex …” change to “… 62% did not use a condom at last sex …”

**Authors’ Response**: Changed (lines 211-212).

- In Table 1 “ first sex bonfire age 15” should read “First sex before age 15 years”

**Authors’ Response**: Edited.

- Line 205. “…among those aged 15-24…” should be “…. Among those aged 15-24 years…”

**Authors’ Response**: Edited (line 233).

- Repetition - line 195 and Line 196 (“…..had sex before age 15 (6.8%)…)

**Authors’ Response**: repeated content deleted (line 225).

- Line 309 “….receive….” is that supposed to be “….achieve...”

**Authors’ Response**: “.. receive ..”. Sentence edited to read ‘receive the continuum of HIV care and treatment services’... (line 356)

- Table 2: missing “n” for viral suppression for the subgroup “did not use a condom”

**Authors’ Response**: Correct. “n” is included now

- Line 253: Is figure 5 (Appendix 1), prepared as part of this analysis or is reference? In either case, it needs to be clearly described in the methods section.

**Authors’ Response**: The figures were created by the authors using data from sources other than EPHIA. Given the comment/suggestion form the Editor, we have, as explained above, opted to remove the appendices.

Reviewer #2: The authors report findings of a large household survey conducted in urban areas of Ethiopia. The survey collected socio-demographic and behavioral indicators as well as self-reported HIV and treatment status information via questionnaires, and assessed HIV status, antiretroviral treatment (ART) use, and viral load suppression empirically based on blood samples from consenting participants. The authors report estimates of HIV prevalence, as well as proportions of people living with HIV who know their status, are on ART, and/or who are virally suppressed. These methods are consistent with other Population-based HIV Impact Assessment surveys done in sub-Saharan Africa, are sound, and provide data appropriate for these analyses.

This manuscript is an important contribution to the research literature, as well as for understanding the HIV epidemic in urban areas of Ethiopia. I believe the manuscript itself would benefit from additional detail around the methods, and from copy-editing to improve clarity. I have discussed these in specific comments below.

Major comments:
1. I would like to see more care that these results are not presented as nationally representative, e.g. at lines 32 and 47-49, given the urban scope of the survey. I appreciate that the urban scope is discussed as a limitation; please include the rationale for this choice in the Methods section as well. Line 88-89 (“a reliable nationally representative number of PLHIV for HIV programmatic planning was lacking”) draws particular attention to this limitation, so it would help to address it earlier.

**Authors’ Response**: The urban focus (rationale) is now emphasized upfront in the methods section. As explained above, language in the limitation section is tempered.

2. The role of the weights in producing these estimates is not clear (e.g., line 141). Please provide a bit more detail about the survey design and what these weights account for.

**Authors’ Response**: Details on the weighting are now added (lines149-157)

3. There is a big drop-off from the 25,416 selected household members to the 21,560 considered eligible (Figure 1). Please elaborate on the eligibility criteria a bit more, and the potential limitations those might impose. For example, how many potential respondents were ineligible because they did not speak one of the survey languages (line 155), and might their exclusion have biased estimates?

**Authors’ Response**: Details on eligibility and numbers are now added (lines 192-201) and shown in Figure 1.

4. In the results section and especially the figures and tables, please make sure the denominators are clear when looking at the “absolute” cascade (percentages of all PLHIV) vs. the “conditional” cascade (e.g. viral suppression among PLHIV on ART).

**Authors’ Response**: Indicated in Table 2 that the estimates are conditional. Indicated in the text (line 276) that the denominator for the estimates in Table 3 are conditional. Denominators for Figures 3 and 4 are conditional (indicated in the captions)

Unconditional (absolute) estimates are only shown in Figure 2. This is clearly indicated in the caption of the figure and in the corresponding description (line 235).

5. There are a couple places where it seems overly strong to claim that there are no differences in estimates, when differences in estimates apparently exist but perhaps did not reach statistical significance. For example, at line 228 the authors report that the “second 90 does not differ by age, gender, or administrative region”, but there are some numerical differences in these estimates in Table 2. This also applies to the third 90 at line 234-235.

**Authors’ Response**: Revised to show the differences in the estimates of the associations, while emphasizing that the differences did not achieve statical significance - lines 252-258 for second 90, and lines 259-266.for the third 90.

6. The 97% ART coverage among PLHIV who know their status is praiseworthy, but I think it would be appropriate to temper the claim that the second 90 target has been achieved (Line 278). That situation may change as efforts to reach the first 90 target continue. The first two 90’s together would imply that at least 81% of PLHIV are on ART, in comparison to the 77% reported in Figure 1, which owes to the gap in achievement of the first 90. Efforts to maintain high ART coverage among those aware must be sustained as HIV status awareness improves.

**Authors’ Response**: We have tempered the claim (lines 331-332). We have also incorporated the implications of increases in HIV status awareness will have on the need to maintain ART coverage (lines 332-333).

Minor comments:
1. Line 27: Missing punctuation before “some believed the targets were not achievable”?

**Authors’ Response**: Edited (line 27).

2. Line 29-31: Perhaps the sentence starting “The overall cascade of HIV-positive people […]” should be in the Methods section.

**Authors’ Response**: Deleted. Content of the sentence well addressed in the methods section.

3. Line 37: For context, it would help to list which ARVs were screened for in the methods section, and how those lined up with ARVs commonly in-use in Ethiopia when the survey was conducted.

**Authors’ Response**: The ARVs screened and their use status at the time of the survey are added (lines 34-35)

4. Line 40-46: Please report uncertainty bounds about the estimates.

**Authors’ Response**: Uncertainty bounds are now included for all estimates (in results sections of the methods (lines 39-47, 217-228, and 245-266)

5. Line 42: “ARV” has not been defined.

**Authors’ Response**: Corrected - expanded for used here (line 41)**.**

6. Line 44-46: Since the 90-90-90 targets have not been formally defined in the preceding text, “variation in the achievement of the first 90 target” may be unclear to some readers. Please either define the three components of the 90-90-90 targets or describe this more directly ("variation in knowledge of HIV-positive status").

**Authors’ Response**: Opted for the second option of describing it more directly (line 50).

7. Line 55: “United Nations Programme of HIV/AIDS” should be “the Joint United Nations Programme on HIV/AIDS” as at line 25.

**Authors’ Response**: Corrected (line 55).

8. Line 71-82: Please check the references in this section. For example, reference (8) pertains to TB and does not seem to support the claims here regarding declines in HIV incidence and mortality.

**Authors’ Response**: Right. Corrections are made in this section. Ref #8 deleted, and Ref#10 moved here as this is the correct reference (lines 72-83).

Changes made in the references and reasons for the changes are given in detail on page 8 above

9. Line 81-82: The sentence starting “Treatment failure[…]” seems a bit disconnected from the rest of the paragraph.

**Authors’ Response**: A new ref on barriers is added, and the section revised for better flow, including the linking of the sentence on treatment failure with the rest of the paragraph (lines 81-83).

10. Line 96: “countries” should be “country’s”

**Authors’ Response**: Corrected and the structure of the sentence revised (line 95).

11. Line 114: Please clarify whether these eleven regions refer to the nine regional states and two city administrations?

**Authors’ Response**: This is clearly stated (nine regional states and two city administrations) earlier under ‘*Survey setting and population’* in the methods section (line 98-99) and consistently across the manuscript and in the rest of the manuscript, where this is required.

12. Line 139: Please close or remove the unmatched parentheses here and throughout the text and tables.

**Authors’ Response**: Done (line 146) and across the manuscript.

13. Line 170: Please define “EA”.

**Authors’ Response**: Defined (line 194).

14. Table 1: (a) There seems to be a typo for the total HIV-negative by sex (n=46205). (b) Should “First sex bonfire age 15” be “First sex before age 15”?

**Authors’ Response**: Both typos are corrected in Table 1.

15. Figure 2: (a) There should not be confidence intervals on the UNAIDS targets. (b) Please lighten the bar color for the survey-based estimates, as the lower limits of the uncertainty bounds are barely visible.

**Authors’ Response**: (a) Corrected (CIs is removed) (b) colour of the bar is lightened.

16. Table 2: The sample size for viral suppression among those who did not use a condom is missing.

**Authors’ Response**: Corrected – sample size for VLS is added in Table 2.

17. Line 231: Should “condom use in the last 24 hours” be “condom use at last sex in the last 12 months”?

**Authors’ Response**: Yes, corrected (line 257-258).

18. Figures 5-7: I believe these estimates come from different time points? Please clarify.

**Authors’ Response**: Figures 5-7 (Appendices 1-3) are removed as they have limited importance to the objectives (also describe above) on pages 4, 8 and 9).

19. Line 287-88: “[…] two-thirds of all HIV transmissions were estimated to be from adult men to women. ”Please provide a citation for this.

**Authors’ Response**: A citation (ref#40) added (line 342).

20. I spotted a couple issues in the references, please review: (a) Line 354: The title is incorrect. (b) Line 416: The author (UNAIDS) is garbled.

**Authors’ Response**: All references, including the newly added ones are checked for completeness and accuracy based on the Vancouver style.

6. PLOS authors have the option to publish the peer review history of their article ([what does this mean?](https://journals.plos.org/plosone/s/editorial-and-peer-review-process#loc-peer-review-history)). If published, this will include your full peer review and any attached files.


If you choose “no”, your identity will remain anonymous but your review may still be made public.


**Do you want your identity to be public for this peer review?** For information about this choice, including consent withdrawal, please see our [Privacy Policy](https://www.plos.org/privacy-policy).

Reviewer #1: No

Reviewer #2: **Yes:** Robert Glaubius

[NOTE: If reviewer comments were submitted as an attachment file, they will be attached to this email and accessible via the submission site. Please log into your account, locate the manuscript record, and check for the action link "View Attachments". If this link does not appear, there are no attachment files.]

While revising your submission, please upload your figure files to the Preflight Analysis and Conversion Engine (PACE) digital diagnostic tool, <https://pacev2.apexcovantage.com/>. PACE helps ensure that figures meet PLOS requirements. To use PACE, you must first register as a user. Registration is free. Then, login and navigate to the UPLOAD tab, where you will find detailed instructions on how to use the tool. If you encounter any issues or have any questions when using PACE, please email PLOS at [figures@plos.org](mailto:figures@plos.org). Please note that Supporting Information files do not need this step.

*In compliance with data protection regulations, you may request that we remove your personal registration details at any time.* [*(Remove my information/details)*](https://www.editorialmanager.com/pone/login.asp?a=r)*. Please contact the publication office if you have any questions.*

|  | ReplyForward |
| --- | --- |
