# Supplementary material for: Progress towards controlling the HIV epidemic in urban Ethiopia: Findings from the 2017–2018 Ethiopia population-based HIV impact assessment survey
Source: PLoS One. 2022 Feb 25;17(2):e0264441. doi: 10.1371/journal.pone.0264441 (PMC8880883; doi:10.1371/journal.pone.0264441)
Supplement: Attachment — Submitted filename: Response to Reviewers.docx [file pone.0264441.s003.docx]

PLOS ONE Decision: Revision required [PONE-D-21-26375R1] - [EMID:457c4fee2104e391]

Inbox

| **PLOS ONE <em@editorialmanager.com>** | 5:25 PM (20 minutes ago) |  |    |
| --- | --- | --- | --- |
| to me  | | |  |

PONE-D-21-26375R1

Progress towards controlling the HIV epidemic in urban Ethiopia: Findings from the 2017-2018 Ethiopia population-based HIV impact assessment survey

PLOS ONE

Dear Dr. Lulseged,

Thank you for submitting your manuscript to PLOS ONE. After careful consideration, we feel that it has merit but does not yet fully meet PLOS ONE’s publication criteria as it currently stands. Therefore, we invite you to submit a revised version of the manuscript that addresses the points raised during the review process.

Both reviewers have responded favorably to the changes made in Revision #1.  The article seems on its way toward publication in PLOS ONE.  All that remains is to address the remaining concerns of Reviewer #2. Provided that these primarily straightforward concerns are addressed in the next version, I do not anticipate needing to send the article back out to the reviewers.

Please submit your revised manuscript by Mar 07 2022 11:59PM. If you will need more time than this to complete your revisions, please reply to this message or contact the journal office at [plosone@plos.org](mailto:plosone@plos.org). When you're ready to submit your revision, log on to <https://www.editorialmanager.com/pone/> and select the 'Submissions Needing Revision' folder to locate your manuscript file.

Please include the following items when submitting your revised manuscript:

- A rebuttal letter that responds to each point raised by the academic editor and reviewer(s). You should upload this letter as a separate file labeled 'Response to Reviewers'.
- A marked-up copy of your manuscript that highlights changes made to the original version. You should upload this as a separate file labeled 'Revised Manuscript with Track Changes'.
- An unmarked version of your revised paper without tracked changes. You should upload this as a separate file labeled 'Manuscript'.

If you would like to make changes to your financial disclosure, please include your updated statement in your cover letter. Guidelines for resubmitting your figure files are available below the reviewer comments at the end of this letter.

If applicable, we recommend that you deposit your laboratory protocols in [protocols.io](http://protocols.io/) to enhance the reproducibility of your results. Protocols.io assigns your protocol its own identifier (DOI) so that it can be cited independently in the future. For instructions see: <https://journals.plos.org/plosone/s/submission-guidelines#loc-laboratory-protocols>. Additionally, PLOS ONE offers an option for publishing peer-reviewed Lab Protocol articles, which describe protocols hosted on [protocols.io](http://protocols.io/). Read more information on sharing protocols at <https://plos.org/protocols?utm_medium=editorial-email&utm_source=authorletters&utm_campaign=protocols>.

We look forward to receiving your revised manuscript.

Kind regards,

David Gerberry, Ph.D.
Academic Editor
PLOS ONE

Journal Requirements:

Please review your reference list to ensure that it is complete and correct. If you have cited papers that have been retracted, please include the rationale for doing so in the manuscript text, or remove these references and replace them with relevant current references. Any changes to the reference list should be mentioned in the rebuttal letter that accompanies your revised manuscript. If you need to cite a retracted article, indicate the article’s retracted status in the References list and also include a citation and full reference for the retraction notice.

[Note: HTML markup is below. Please do not edit.]

Reviewers' comments:

Reviewer's Responses to Questions

**Comments to the Author**

1. If the authors have adequately addressed your comments raised in a previous round of review and you feel that this manuscript is now acceptable for publication, you may indicate that here to bypass the “Comments to the Author” section, enter your conflict of interest statement in the “Confidential to Editor” section, and submit your "Accept" recommendation.

Reviewer #1: All comments have been addressed

Reviewer #2: (No Response)

2. Is the manuscript technically sound, and do the data support the conclusions?

The manuscript must describe a technically sound piece of scientific research with data that supports the conclusions. Experiments must have been conducted rigorously, with appropriate controls, replication, and sample sizes. The conclusions must be drawn appropriately based on the data presented.

Reviewer #1: Yes

Reviewer #2: Yes

3. Has the statistical analysis been performed appropriately and rigorously?

Reviewer #1: Yes

Reviewer #2: I Don't Know

4. Have the authors made all data underlying the findings in their manuscript fully available?

The [PLOS Data policy](http://www.plosone.org/static/policies.action#sharing) requires authors to make all data underlying the findings described in their manuscript fully available without restriction, with rare exception (please refer to the Data Availability Statement in the manuscript PDF file). The data should be provided as part of the manuscript or its supporting information, or deposited to a public repository. For example, in addition to summary statistics, the data points behind means, medians and variance measures should be available. If there are restrictions on publicly sharing data—e.g. participant privacy or use of data from a third party—those must be specified.

Reviewer #1: Yes

Reviewer #2: Yes

5. Is the manuscript presented in an intelligible fashion and written in standard English?

PLOS ONE does not copyedit accepted manuscripts, so the language in submitted articles must be clear, correct, and unambiguous. Any typographical or grammatical errors should be corrected at revision, so please note any specific errors here.

Reviewer #1: Yes

Reviewer #2: Yes

6. Review Comments to the Author

Please use the space provided to explain your answers to the questions above. You may also include additional comments for the author, including concerns about dual publication, research ethics, or publication ethics. (Please upload your review as an attachment if it exceeds 20,000 characters)

Reviewer #1: The Authors have now applied a more appropriate statistical analysis methodology, presented and explained the results well to my satisfaction. The explanations are now clearer and easy to follow.

The authors have done a commendable Job and addressed all my comments satisfactorily. I congratulate them upon this. I have a few minor comments here below.

Line 42 to line 45: “Awareness Females ….” Statement not clear, review and correct

**Authors’ Response**: Reviewed and corrected (lines 42-45 now).

Line 77:”… prevalence of HIV infection in all aged,” replace aged with “age groups”

**Authors’ Response**: Corrected (line 78).

Line 85: “… health survey (EDHS) was supplemented by ….” Replace with “… health survey (EDHS), supplemented by ….”

**Authors’ Response**: Replaced (line 86).

Line 321: “…the implication high risk …” replace with “….the implication of high risk…”

**Authors’ Response**: Replaced (line 322)

Line 305; “… 79% knew that they were HIV positive status” drop the word status

**Authors’ Response**: Dropped (line 306).

Line 345- 350: the conclusion on the paragraph does not reflect the result in the paragraph, please review. i.e if age and gender were not statistically significant, what is the basis of targeting young people and men as you have stated in the paragraph

**Authors’ Response**: Lines 351-353 are edited by indicating the emphasis here is “increasing awareness”. This is now included.

Reviewer #2: The authors have largely addressed each of my comments. Most of my comments below are on copy-editing issues rather than methodological concerns, though I do have some questions regarding the new multivariate regression analysis.

1. I appreciate that the authors are now more explicit about "unconditional" and "conditional" estimates and targets. These terms should be defined in the methods section, or replaced with more direct statements, as has been done in Table 3 (e.g. "Virally suppressed among HIV+ participants on ART").

**Authors’ Response**: Defined in the methods section (lines 146-151)

2. I also appreciate the new multivariate regression analysis. I answered "I don't know" with regards to the appropriateness and rigor of the approach because I feel we need a bit more information here. Could you please explain how you chose which explanatory variables to include? Was age modeled as a continuous variable or a categorical variable? Either is valid, but if modeled categorically, that might have diluted any age effects so it seems worth mentioning. Finally, Table 3 includes education level but Table 2 does not. Could that be added to Table 2 to provide more context?

**Authors’ Response**:

- For the multivariate regression analysis, all variable achieving a significance level of p<0.2 were selected. This explanation is now included (lines 168-169).
- Age was modelled as a categorical variable. The research question was to assess the association between age group and the three-90 outcomes (Table 3).
- Education is now added to Table 2.

3. Please review Table 1 carefully for accuracy and clarity:
3.a. Total rows for administrative region and sex incorrectly state the 95% CI for HIV prevalence as ".8-3.3"; this is reported as "2.8-3.3" at line 218.

**Authors’ Response**: These are now corrected in Table 1.

3.b. Under "First sex before age 15", percentages HIV-positive and HIV-negative are missing.

**Authors’ Response**: Included.

3.c. HIV prevalence among those never tested cannot be right, "1 9 (0.7-1.3)", if 99.0% of those never tested were HIV-negative.

**Authors’ Response**: Yes there is an error here. Corrected

3.d. The 95% CI for the % of never-tested who were HIV-negative seems far too wide (8.7-99.0).

**Authors’ Response**: Yes there is an error here. Corrected

3.e. There are several typos, like square brackets sometimes used in place of parentheses, duplicate parentheses ("))"), and inconsistent use of commas and percentage signs. These may be minor individually, but together suggest that there could be other undetected errors in the results too.

**Authors’ Response**: Manuscript edited for typos, including for those indicated in the comment.

4. The authors have improved many of the copy-editing issues identified in the previous round, but there are some issues remaining:
4.a Line 42:. "Awareness Females about HIV-positive[...]" perhaps should be "Awareness of HIV-positive status[...]"?

**Authors’ Response**: Edited (line 42).

4.b. Lines 166-168 repeat lines 97-99; these could be merged.

**Authors’ Response**: Merged. As suggested by one of the reviewers in a previous comment to bring this right to the beginning of the Methods Section, text in lines 167-169 is deleted, and text in lines 98-100 edited to accommodate text in both (lines .

4.c. Line 194-201: The paragraph duplicates the number eligible among household members identified (lines 192 & 195) and interviewed among eligible (lines 195 & 199). Perhaps this paragraph could be revised to eliminate redundancy?

**Authors’ Response**: Revised as suggested (196-203).

4.d. Line 207: "[...]34.9% were 1 in the[...]". Please remove "1".

**Authors’ Response**: Removed 1 (209 now).

4.e. Line 223: "[...]and food-insecure 95% CI (5.0% [95% CI: 3.7-6.8])". Please remove the first "95% CI".

**Authors’ Response**: Removed (line 225 now).

4.f. Line 223-224: "[...]than the categories[...]" should be "[...]than in the other categories[...]"

**Authors’ Response**: Edited as suggested (lines 225-226 now)

4.g. Line 233: Please consider changing "77% of them on ART" to "77% were on ART"

**Authors’ Response**: Done (line 235 now)

4.h. Line 233: Please consider changing "had viral suppression" to "were virally suppressed" or "had suppressed viral loads".

**Authors’ Response**: Changed (line 235 now)

4.i. Line 246: You might change "[...]adjusted for ARV detection." to "[...]when adjusted for ARV detection."

**Authors’ Response**: Changed (line 248 now)

4.j. Line 266: I think this is missing a period, and perhaps there should not be a paragraph break here?

**Authors’ Response**: Edited (268 now)

4.k. Figure 4: You might remove "based on ARV" since the caption states that these estimates are ARV-adjusted.

**Authors’ Response**: Right; "based on ARV" removed.

7. PLOS authors have the option to publish the peer review history of their article ([what does this mean?](https://journals.plos.org/plosone/s/editorial-and-peer-review-process#loc-peer-review-history)). If published, this will inclu235 now)de your full peer review and any attached files.


If you choose “no”, your identity will remain anonymous but your review may still be made public.


**Do you want your identity to be public for this peer review?** For information about this choice, including consent withdrawal, please see our [Privacy Policy](https://www.plos.org/privacy-policy).

Reviewer #1: **Yes:** Joseph Ouma

Reviewer #2: No

[NOTE: If reviewer comments were submitted as an attachment file, they will be attached to this email and accessible via the submission site. Please log into your account, locate the manuscript record, and check for the action link "View Attachments". If this link does not appear, there are no attachment files.]

While revising your submission, please upload your figure files to the Preflight Analysis and Conversion Engine (PACE) digital diagnostic tool, <https://pacev2.apexcovantage.com/>. PACE helps ensure that figures meet PLOS requirements. To use PACE, you must first register as a user. Registration is free. Then, login and navigate to the UPLOAD tab, where you will find detailed instructions on how to use the tool. If you encounter any issues or have any questions when using PACE, please email PLOS at [figures@plos.org](mailto:figures@plos.org). Please note that Supporting Information files do not need this step.

*In compliance with data protection regulations, you may request that we remove your personal registration details at any time.* [*(Remove my information/details)*](https://www.editorialmanager.com/pone/login.asp?a=r)*. Please contact the publication office if you have any questions.*

Attachments area
